# Assessment of Mechanical, Thermal and Durability Properties of High-Volume GGBS Blended Concrete Exposed to Cryogenic Conditions

**DOI:** 10.3390/ma14092129

**Published:** 2021-04-22

**Authors:** Giyeol Lee, Okpin Na

**Affiliations:** 1 Department of Landscape Architecture, College of Agriculture and Life Science, Chonnam National University, Gwangju 61186, Korea; gylee@jnu.ac.kr; 2 R&D Division, Hyundai E&C, Yongin-si 16891, Korea

**Keywords:** cryogenic condition, GGBS, compressive strength, thermal conductivity, semi-adiabatic test

## Abstract

The purpose of this study is to suggest the optimum mix design with a high volume of GGBS (Ground Granulated Blast-furnace Slag) replacement and the procedure of the cryogenic test to consider mechanical and thermal properties, and durability performance. To decide the optimum mix design, four mix designs with high-volume of GGBS replacement were suggested, in terms of the slump and retention time. Based on the test results, with respect to the workability and compressive strength, the mixtures with 65% of GGBS (C40-2 and C40-4) were better than the mixtures with 50% and 60% of GGBS (C40-1 and C40-3). After selecting two mixtures, two types of cryogenic test methods were conducted under one-cycle cryogenic condition (Test A) and 50-cycles cryogenic condition (Test B). As a result, in Test A, the compressive strength and elastic modulus of the C40-2 and C40-4 mixtures tended to be decreased over time, because of the volume expansion of ice crystals contained in the capillary pores. In Test B, the mechanical properties of the C40-4 mixture were better than those of the C40-2 mixture, in terms of the reduction rate of compressive strength and elastic modulus. In the view of the heat of hydration, the semi-adiabatic test was conducted. In the results, the C40-4 mixture was better to control the thermal cracks. Thus, the C40-4 mixture would be more suitable for cryogenic concrete and this procedure could be helpful to decide the mixture of cryogenic concrete. In the future, the long-term performance of cryogenic concrete needs to be investigated.

## 1. Introduction

LNG (Liquefied Natural Gas) has been regarded as the most realistic alternative to reduce global warming from petroleum energy because it emits very little sulfurous acid gas (SO_2_) recognized as a major cause of environmental problems. LNG demand is expected to be determined by the climate change response activities of each country around the world. Most of all, Asia is the world’s largest importing region of LNG and accounts for two-thirds of global consumption [1,2,3,4].

Natural gas is cooled to about −163 °C to convert the gaseous gas to a liquid state for storage. Therefore, many kinds of research have been conducted to minimize heat loss and ensure safety against gas leakage [5,6,7].

LNG storage tanks can be largely divided into above-ground, in-ground and under-ground depending on the installation location. According to the type of inner tank, it can be roughly classified into a 9%-nickel steel tank and membrane tank. Based on the definition of NFPA 59 A (2001), BS EN 1473 (1996) and BS 7777 (1993), a 9%-Ni steel tank is classified into single, double and fully containment LNG storage tanks. The full containment LNG storage tank with relatively high safety is a double tank structure in which the inner tank and the outer tank can independently store LNG at cryogenic temperatures [8].

The inner tank stores cryogenic LNG under normal operating conditions and the outer tank is located between 1 m and 2 m from the inner tank and functions as a dike as well as has a function to support the outer roof. The tank has a prestressed concrete outer container with a flexible inner container and insulation supported by an outer tank wall. Prestressed concrete (PC) structures are suitable for storing LNG and their design incorporates special loads and a special performance at cryogenic temperatures [4,8,9,10].

Nevertheless, the inner and outer storage tanks of LNG involve several potential risks. Sudden failure of LNG storage tank is not acceptable, since the escaped liquid would vaporize, mix with air and form an explosive cloud. The explosions or fires resulting from such an event could lead to an unacceptable loss of life and damage to the plant and environment. To reduce the potential risks and safety issues of a concrete storage tank, design guidance is given in BS 7777, NFPA 95A and BS EN 1473 [10,11]. Moreover, Jeon et al. (2003) studied the liquid tightness design associated with the cryogenic temperature under the emergency condition of LNG leakage [6]. After then, Jean et al. (2004) focused on the major factors deciding the shape of the large LNG tank [12]. Hoyle (2013) in Chevron presented the modular design of the precast concrete outer wall, instead of the in-situ concrete wall for the full containment storage tank. It concluded that this modular concept could replace the 9%-nickel steel with concrete and reduced the material cost and construction time [13]. Based on the composite concrete cryogenic tank (C3T) of Chevron, Jeon et al. (2014) and Jo et al. (2015) also studied the precast concrete module with outer liners to shorten the construction period [14,15]. Even though the concrete module could make construction time to be saved, the connection between concrete panels and countermeasures of emergency leakage should be supported with sufficient researches.

**Figure 1:**
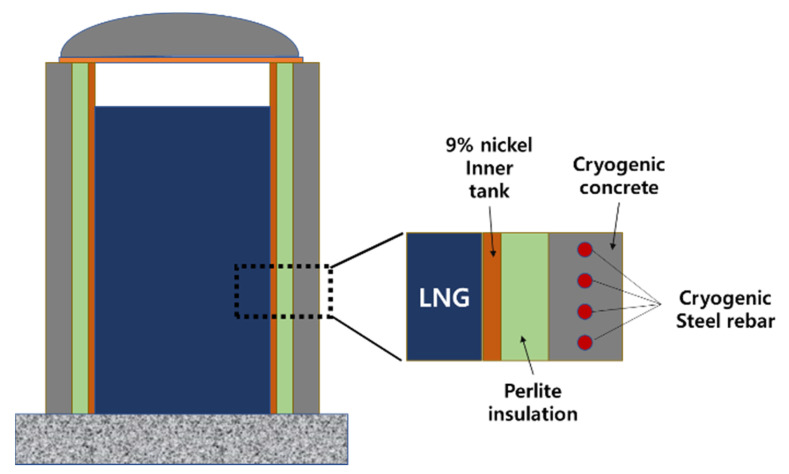
Full containment LNG storage tank.

Generally, impermeable insulations such as PUF (Poly Urethane Foam) have been located between the inner tank and outer concrete wall to prevent direct contact as demonstrated in Figure 1. Recently, to remove the impermeable insulation, cryogenic rebars as reinforcement at the inner surface of the concrete wall were partially used. Yoon (2012) suggested the application of cryogenic rebar to LNG storage outer tank [16]. Cryogenic steel rebar is used to prevent the brittle failure of concrete outer wall when the leakage of LNG occurs. Cryogenic steel rebar is a specially-designed concrete reinforcing steel for cryogenic applications and is suitable for use in storage tanks with temperatures down to −170 °C in accordance with EN 14620-3:2006. In fact, cryogenic rebar has been manufactured by Commercial Metals Company (CMC) in USA and ArcelorMittal in Luxembourg City.

Concrete used as the outer shell can be directly exposed to the LNG leakage and the inner surface of outer concrete can be cooled lower than −165 °C of cryogenic temperature. The concrete used to contain the liquefied natural gas must withstand sub-arctic temperatures as low as −165 °C and is called “cryogenic concrete”. However, concrete behavior at cryogenic conditions has been not elucidated. Kogbara et al. (2013) reviewed the concrete properties under cryogenic temperatures such as permeability, coefficient of thermal expansion (CTE), tensile strength, bonding strength, compressive strength and so on [17]. Kogbara et al. (2014) investigated the damaged microstructure of concrete due to cryogenic temperature including the effect of aggregate type and introduced the design method of a damage-resistance cryogenic concrete. To demonstrate the damage effects before and after freezing, acoustic emission (AE) and X-ray computed tomography (XRCT) methods were employed. The results indicated that microcracking resistance of concrete after the cryogenic condition was very related to the type of coarse aggregate [18]. Dahmari et al. (2007) mentioned the basic cause of concrete failure under cyclic freezing resulted from the transition to ice from free water in the pores and lead to the reduction in strength and structural damage [19]. Especially, Kwak et al. (2008) studied to measure the change in mechanical properties of concrete exposed to a specific temperature range from −20 to −60 °C [7]. To reveal the fracture properties at temperatures ranging from 20 to −170 °C, Rocco et al. (2001) conducted three-point bending tests on notched beams and determined the fracture parameters with the cohesive crack model, in terms of tensile strength, fracture energy, softening curve (stress vs. crack opening), characteristic length and modulus of elasticity [20]. Recently, for developing outer concrete, Kim et al. (2018) investigated the flexural and cracking behavior of ultra-high-performance fiber-reinforced concrete (UHPFRC) before and after exposure to cryogenic temperatures through four-point bending tests. The test results indicated that UHPFRC had higher resistance to microcrack formation and better flexural performance rather than normal concrete [21]. Moreover, Mazur et al. (2019) also carried out laboratory tests to reveal the negative effect under low temperature and suggest the improved ways of mix design with respect to decease in *w/c* ratio, type of cement and aggregate and use of aeration admixture [22].

As shown in Figure 1, concrete outer wall in LNG tank is generally designed as mass concrete with 1 m thick and more and most of LNG tanks are located in coastal area. Thus, this concrete outer wall can be easily exposed to chloride-rich environment. To enhance the concrete durability in corrosive environment, high-volume of GGBS (Ground Granulated Blast-furnace Slag) should be added. Based on ACI 233 and some references, basically, more than 50% of GGBS replacement in concrete mixture has an influence on the improvement of the durability and the reduction of the heat of hydration in mass concrete [23,24,25,26,27]. Rashad et al. (2017) and Rachel et al. (2019) investigated the mechanical and durability properties of the high-volume GGBS mixture with metakaolin and flyash. Even though the replacement of GGBS increased, test results of RCPT (Rapid Chloride Permeability Test), sorptivity and water permeability were lower than those of conventional concrete mixture [25,26]. Recently, Lee et al. (2020) evaluated the optimal CaO content range to secure the durability performance. As a result, the optimal CaO content was within range of about 55% and the replacement ratio of GGBS was about 50% [27]. Therefore, high-volume of GGBS replacement would be necessary to improve the durability performance of concrete outer tank installed on the coastal area.

Despite the progress of many types of research about the properties of cryogenic concrete, there are some limitations of the researches using the composition of high-volume GGBS binder in mix design. In addition, sufficient research results about the practical procedure of cryogenic tests have not been provided significantly with focus on the concrete properties. Therefore, the purpose of this study is to suggest the optimum mix design with a high volume of GGBS replacement and the procedure of the cryogenic test to consider mechanical and thermal properties, and durability performance based on the review of ACI 376 [28].

Above all, ACI 376 was reviewed to define the investigation items about mechanical and durability properties under cryogenic environment. Then, all raw materials used in mix design were tested to compare the test results with requirements in ASTM and BS codes. Particularly in this study, high-volume of GGBS and air entrainer admixture were used to the control of heat of hydration and durability for the increase of freeze-thawing resistance in accordance with emergency condition of LNG leakage. Two types of cryogenic conditions were employed, and various specimens were tested to measure the mechanical, thermal and durability properties. Finally, with mock-up specimens, productivity and semi-adiabatic tests were carried out.

## 2. Experimental Plan

### 2.1. Materials and Mix Design

All materials used in concrete followed the related standards in Table 1. Cement was combined with Ground Granulated Blast-furnace Slag (GGBS) in conformity to ASTM C 595 [29]. Table 2 shows the requirements for cement from ASTM C 150 and all test results satisfied with the requirements are provided by the supplier [30].

In order to achieve the strength and durability of the concrete, the replacement of GGBS as a mineral admixture is necessary. GGBS decreases a permeability of concrete and improves chemical resistance such as chlorides and sulfates. It also reduces the heat of hydration related to Delayed Ettringite Formation (DEF). The slag constituent shall not exceed 70% of the mass of total cementitious material in the concrete mix. Table 3 shows the requirements for GGBS from ASTM C 989 and all test results satisfy the requirements [31].

**Figure 2:**
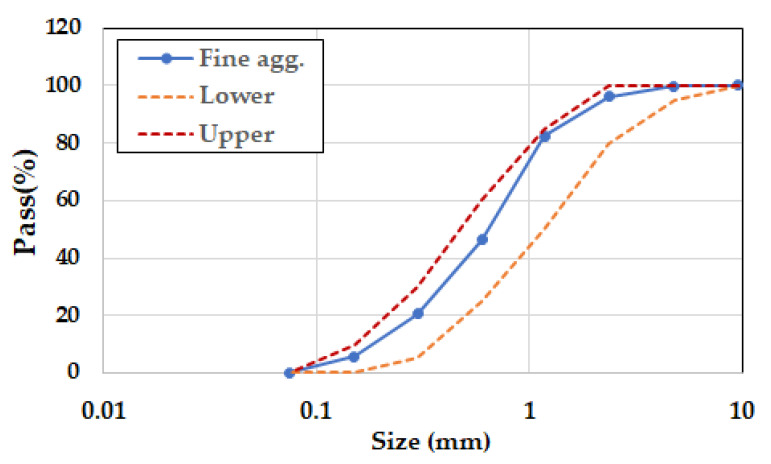
Grading of fine aggregate.

Unwashed original sand contains many fine particles less than the sieve number 200 (0.075 mm) which induce more water and admixture consumption and then weaken the strength and durability of concrete. Sand as fine aggregate was washed at the washing plant before supplied for the test. The grading and the requirement of fine aggregates are shown in Figure 2 and Table 4, respectively. Coarse aggregates were washed gravels or crushed stones in accordance with Table 5. Coarse aggregates were combined with two types of single size, 20 mm and 10 mm and supplied from local providers. In Table 5, all test results for coarse aggregate were compared with the requirements.

In order to improve workability, strength and setting time, a high range and retarding super-plasticizer as a chemical admixture was used, complying with ASTM C 494. Air entrainer admixture shall comply with ASTM C 260. Micro-air 100 as an air entrainer admixture was used for improving a freeze-thaw resistance under a cryogenic environment. Mixing water was used without oil, acid, alkaline and organic matters or deleterious substances, complying with ASTM C 94 as shown in Table 6.

### 2.2. Mix Design for Cryogenic Concrete

The specified compressive strength of cryogenic concrete was 40 MPa and target compressive strength was 50 MPa which was determined by adding 10 MPa to the specified compressive strength due to variations in materials, operations and testing. The maximum size of aggregate was 20 mm. The target slump and slump flow were 220 ± 25 mm and 620 ± 75 mm, respectively. The water/binder ratio was selected with 0.28, and total cementitious content was varied between 475 kg/m^3^ to 495 kg/m^3^. Water content was started from 128 to 133 kg/m^3^ for cryogenic concrete. For water reduction, two types of high-range water-reducing chemical admixtures were applied: Daracem 208 (GCP applied technologies, Cambridge, MA, USA) as naphthalene type and Baxel PC 650 (Baxel, Sharq, Kuwait) as polycarboxylate type. Air content of 4 ± 1.5% was also achieved using a proper air-entraining admixture. Mix proportions are listed as shown in Table 7. Concrete materials were mixed by following ASTM C 192.

### 2.3. Preparation of SPECIMENS and Test Methods

For fresh concrete, air content and retention time of concrete slump were measured. For hardened concrete, mechanical properties such as compressive strength and elastic modulus were carried out. A total of 15 specimens were prepared in each mixture including reserved samples as shown in Table 8.

For one-time cryogenic test (Test A method), a total of 38 specimens were prepared as referred to Table 9. Test A method consisted of five specified tests: compressive and tensile strength, elastic modulus, thermal expansion coefficient and thermal conductivity. Each test was conducted under four different temperature conditions. For freeze-thaw cyclic test (Test B method), a total of 12 specimens were cast as referred to Table 10. Test B method was conducted to investigate the compressive strength and elastic modulus after 50-times freeze-thaw cycles.

### 2.4. Cryogenic Test Methods

According to ACI 376, cryogenic concrete should be assessed the material properties as follows: compressive strength, elastic modulus, poisson’s ratio, thermal conductivity and durability such as resistance to cycles of freezing and thawing [28]. With taking all properties into consideration, cryogenic tests were performed according to the procedure and criteria of one cycle of cryogenic temperature condition up to −196 °C (Test A method) and cyclic temperature condition between 5–−20 °C (Test B method).

#### 2.4.1. Test A Method: One Cycle of Cryogenic Temperature Condition Up to −196 °C

**Figure 3:**
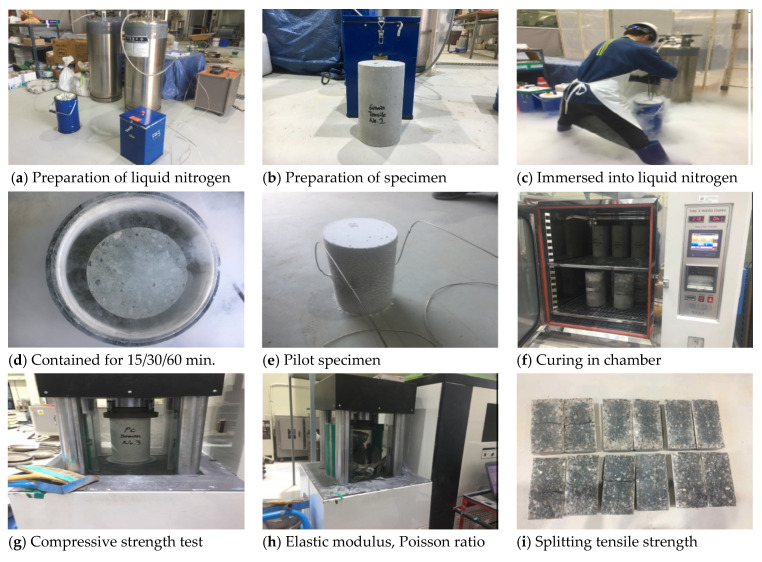

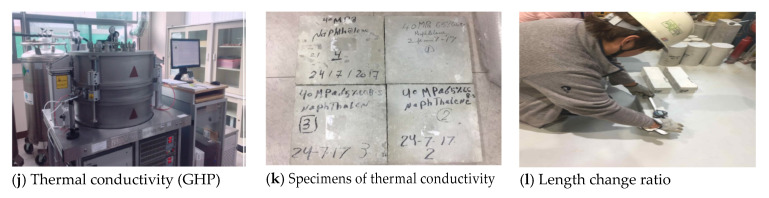
Test A method under cryogenic condition.

The test specimens were subject to a single cycle at very low temperature and subsequently tested. The test results were compared with the concrete characteristic strength (40 MPa). The description and specimens of the test were specified as shown in Table 9. Test specimens were directly immersed into liquid nitrogen for 15, 30 and 60 min. at −196 °C and put out from the insulated storage. The cooled specimens were stored at the curing room (23 °C and RH 95%) where the temperature and moisture could be constantly controlled for 48 h. Reference specimens were kept in the curing room at the same time. After 2 days, the mechanical properties of cryogenic concrete were measured in terms of compressive strength, elastic modulus, poisson ratio, splitting tensile strength, length change for thermal expansion coefficient and thermal conductivity. For measuring the temperature automatically, two thermocouples were installed inside a spare specimen and one sensor was set up outside the spare specimen. The installed locations of thermocouples from the surface of a concrete sample were 25 mm and 75 mm, respectively. The temperature was recorded with a data logger and the temperatures of 15 min, 30 min and 60 min were corresponded to test specimens exposed to the cryogenic condition. The temperature recording was finished before immersing all test specimens into liquid nitrogen. As shown in Figure 3, the cryogenic tests were carried out as follows: compressive strength, tensile strength, elastic modulus, moisture content, length change and thermal conductivity.

#### 2.4.2. Test B Method: Cyclic Temperature Condition between 5–−20 °C

**Figure 4:**
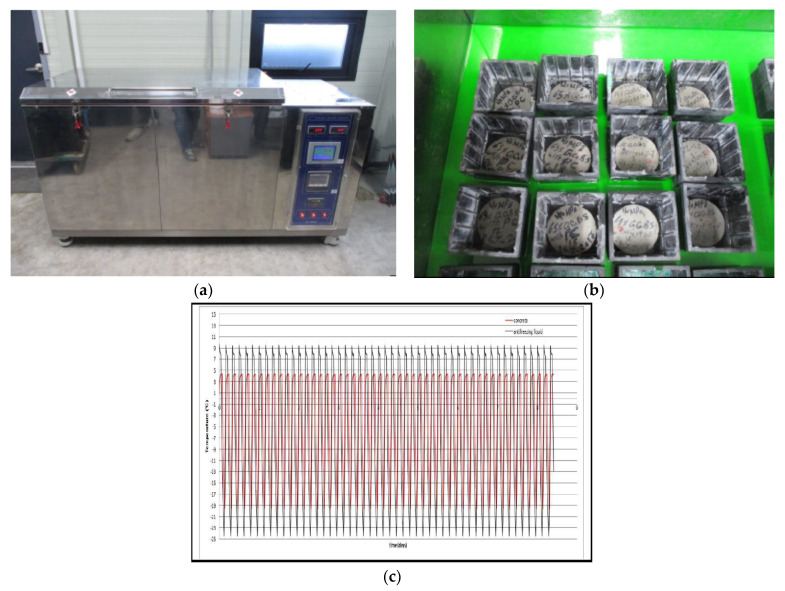
Test B method under cryogenic condition: (**a**) freezing-thawing equipment, (**b**) test set-up with specimens and (**c**) freeze-thaw cycles.

In Figure 4, test specimens were subject to 50 cycles between 5–−20 °C and subsequently tested and test results were compared with the concrete characteristic strength (40 MPa). After finishing freezing and thawing, all samples were maintained in the curing chamber and crushed at the same time as the cooled specimen. The temperature change rate might not be greater than 10 °C per hour. The freeze-thaw cycling test was carried out according to the ASTM C 666. The cooling and heating procedure of the cycle met conditions defined in ASTM C 666. The temperature of specimens was monitored throughout the test used by the thermocouples. The cycled specimens were taken out from the chamber and cycled and un-cycled specimens were crushed at the same time. After finishing the compressive strength tests, the moisture content was determined with the crushed debris dried in an oven for 24 h.

## 3. Test Results and Discussions

### 3.1. Selection of Optimum Mix Design with Slump and Compressive Strength

**Figure 5:**
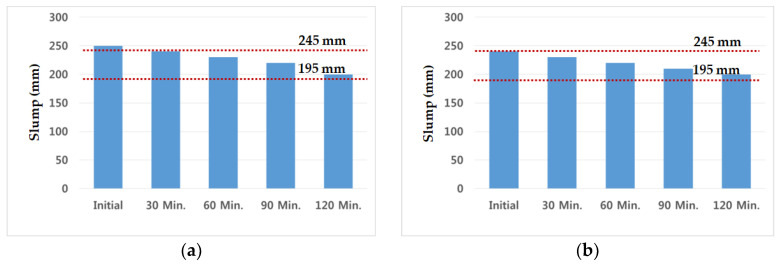
Slump test (**a**) C40-1 (GGBS 50%, Daracem 208) (**b**) C40-2 (GGBS 65%, Daracem 208).

During 120 min after casting, slump tests with admixture Daracem 208 were performed every 30 min, and the testing value of cryogenic concrete was described in Figure 5. The initial slump of the C40-1 mixture exceeded the allowable tolerance, but the other slumps were in the range of it. On the other hand, the slump value of the C40-2 mixture was satisfied during the retention time of 120 min.

**Figure 6:**
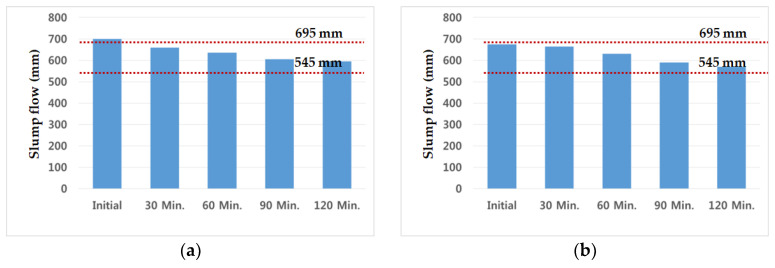
Slump flow test (**a**) C40-3 (GGBS 60%, Baxel PC 650) (**b**) C40-4 (GGBS 65%, Baxel PC 650).

The chemical admixture of C40-3 and C40-4 was used with a polycarboxylate type (Baxel PC 650). The values of slump flow for 120 min were demonstrated in Figure 6. Based on the results, the initial slump flow of the C40-4 mixture with 65% GGBS was satisfied with the tolerance, but the C40-3 mixture with 60% GGBS was not. After 120 min, both of them were in the range of slump flow, 595 mm and 570 mm, respectively. These values were contained within the target range of 620 ± 75 mm. The tolerance of slump flow was within ±75 mm and the C40-4 mixture was more suitable rather than C40-3 one, in terms of retention time and workability.

**Figure 7:**
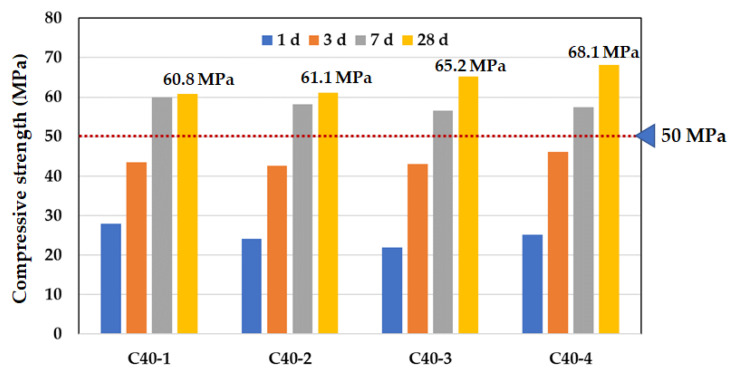
Compressive Strength of concrete mix design over time.

All test samples were cast in accordance with ASTM C 172 and several times of compressive strength tests were conducted for 28 days [49]. The target compressive strength was determined to be more than 50 MPa, including a 10 MPa margin. As shown in Figure 7, the target strengths of all mix designs were sufficiently developed at the age of 7 days. All specimens at the age of 28 days already satisfied the compressive strength of more than 60 MPa. For describing in detail, the binder content of C40-1 and C40-2 was greater than that of C40-3 and C40-4 by 15 kg/m^3^, but the compressive strength of C40-1 and C40-2 was lower than that of C40-3 and C40-4. Therefore, with respect to the workability of fresh concrete, C40-2 and C40-4 were better than C40-1 and C40-3. In view of the development of compressive strength, C40-2 and C40-4 were superior to C40-1 and C40-3. In the next step for cryogenic tests, C40-2 and C40-4 mixture were chosen.

### 3.2. Mechanical, Thermal and Durability Properties under Cryogenic Condition

#### 3.2.1. Mechanical and Thermal Properties after Exposed to a Cryogenic Condition

**Figure 8:**
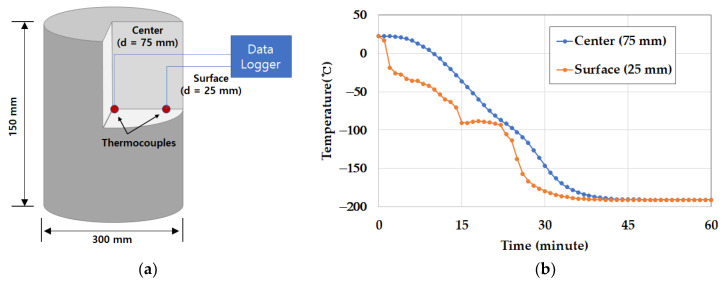
Thermocouple installation and temperature variation: (**a**) sample size and sensor location and (**b**) temperature variation over time.

To figure out the characteristics of the concrete exposed to the cryogenic condition, firstly, two thermocouples in the specimen were installed as shown in Figure 8a. Then, the concrete specimen was put into liquid nitrogen to measure the temperature variation over time. As the result, the initial temperature was started from 22 ± 2 °C and after 45 min, the temperature was dropped down up to −190 ± 2 °C as shown in Figure 8b. The measured temperature variation was regarded as the temperature of the other specimens without thermo-couples. That is, after 15 min, the temperature of the concrete surface went down up to −100 °C. If the specimen was immersed in the liquid nitrogen storage for 15 min, it would be equally considered to be exposed to −100 °C.

In Table 11, the compressive strength and elastic modulus of concrete specimens under cryogenic temperature tended to be decreased over time. When the specimen was frozen under very low temperature, the expansion of ice crystal made some cracks in the capillary pores. Because the compression was loaded to concrete specimens after melting the ice crystal under ambient temperature, compressive strength of the specimens decreased. However, this trend was not the same as mentioned in ACI 376. That is, many studies had shown that compressive strength and elastic modulus increased as the temperature went down [28,50,51,52,53]. As the temperature decreased, the moisture contained in the capillary pores was changed into ice, and the internal structure of the concrete become tight and dense, resulting in an effect of increasing strength. The compressive strength of concrete exposure to cryogenic temperature rose up to about three times compared with ambient temperature. That is, the increment rate in compressive strength of concrete increased as the moisture content increased and the temperature decreased. In particular, below −120 °C, the deviation of compressive strength increased and the increment rate decreased. This was because the volume of ice crystals rapidly reduced around −120 °C or below [52].

**Figure 9:**
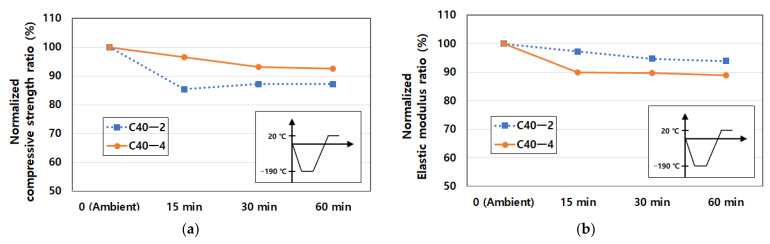
Normalized mechanical properties exposed to cryogenic temperature: (**a**) normalized compressive strength over temperature and (**b**) normalized elastic modulus over temperature.

In Table 11, the reason for the decline of mechanical properties was the expansion of volume. Freezing moisture expanded its volume by about 10%. The expanded volume of ice in the capillary pores caused pressure increase and the excess of the tensile strength of the pore walls impacted on the occurrence of cracks of concrete microstructure. Additionally, the degree of water saturation had a significant effect on the frost resistance of the concrete mix. As shown in Figure 9, the compressive strength and elastic modulus declined over time. That is, in the case of compressive strength, the strength reduction of the C40-2 mixture was about 15% in Figure 9a, but in the case of elastic modulus, 10% of reduction of the C40-4 mixture was observed in Figure 9b. This is because freezing moisture in concrete pores induced the cracks in the pore walls and this cracking resulted in the reduction of mechanical properties [50].

For calculating the coefficient of temperature expansion (CTE), the length change of concrete specimens was measured as shown in Table 12. The CTE of cryogenic concrete was derived as shown in Equation (1) and the CTE of C40-2 and C40-4 was −1.503 and −1.605 × $10^{-6}/{}^{\circ}C$, respectively.

(1) $$C=\frac{\left(R_{h}-R_{l}\right)}{G\cdot \Delta T}$$

where, C = coefficient of linear thermal expansion of the concrete ($10^{-6}/{}^{\circ}C$), $R_{h}$ = length reading at higher temperature (mm), $R_{l}$ = length reading at lower temperature (mm), G = gage length between inserts (mm) and ΔT = difference in temperature of specimen between the two length readings (°C).

**Figure 10:**
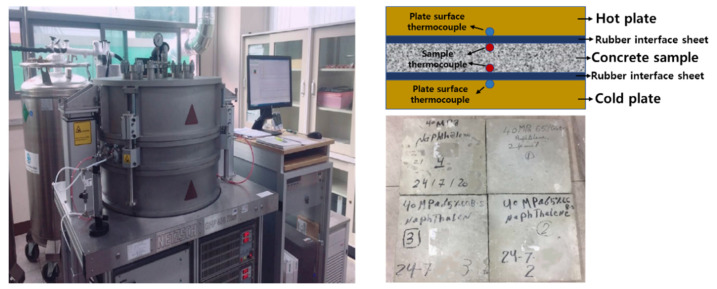
GHP (Guarded Hot Plate) 456 Titan and set-up of the sample, thermal sensors and plate.

For the measure of the thermal conductivity, GHP (Guarded Hot Plate) 456 Titan manufactured by NETZSCH in Selb, Germany was used in Figure 10 and was employed with standardized guarded hot plate technique according to ASTM C 177 [54]. The temperature range of the equipment was −160–(+250) °C and the range of thermal conductivity was 0.003 to 2 W/(m∙K). The two samples of each mix design were prepared and the size of it was a square with 300 mm sides and with 90 mm thick due to the measurement limit of equipment. GHP (Guarded Hot Plate) was based on the absolute measurement method without calibration and correction. The thermal conductivity value resulted in the stationary state and was derived from Equation (2) as follows:

(2) $$\lambda =\frac{\dot{Q}\times d}{2A\times \Delta T}$$

In Equation (1), $\dot{Q}$ is precisely measured total power input into the hot plate, d is average sample thickness, A is measurement area and ΔT is mean temperature difference along the sample.

In ACI 376, the moisture content in concrete have an effect on the thermal conductivity. As temperature goes down, the thermal conductivity rises up linearly. In detail, the thermal conductivity of partially saturated normal-weight concrete increases from approximately 3.2 W/(m∙K) at 25 °C to 4.71 W/(m∙K) at −155 °C [28]. Table 13 demonstrated the thermal conductivity of concrete exposed to ambient and cryogenic temperature.

**Figure 11:**
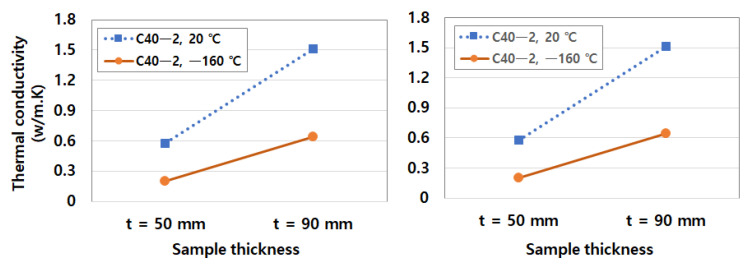
Effect of sample thickness on thermal conductivity.

As a result, the thermal conductivity of C40-2 and C40-4 at ambient temperature (20 °C) was about 1.5 W/(m∙K) and that of C40-2 and C40-4 at very low temperature (−160 °C) was 0.643 and 0.723, respectively. This result was opposite to what ACI 376 mentioned. The factors affecting the thermal conductivity were the ratio of aggregate volume, water-cement ratio, moisture content and curing period. That is, as the volume fraction of aggregate and moisture content increased as well as water-cement ratio and curing period deceased, the thermal conductivity tended to be increased [55]. On the contrary, the test error could be decreased with the thicker specimen. For verifying this tendency, additional thermal conductivity tests were carried out with thinner sample as shown in Figure 11. As the sample thickness was decreased up to 50 mm, the thermal conductivity was down up to 50% or more.

#### 3.2.2. Mechanical Properties after Exposed to Cyclic Low Temperature

**Figure 12:**
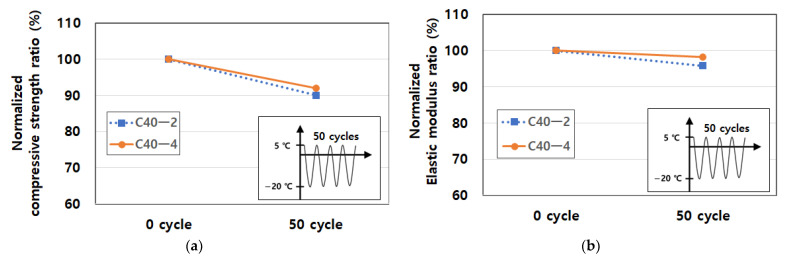
Normalized mechanical properties exposed to cyclic low temperature: (**a**) normalized compressive strength over F-T cycles and (**b**) normalized elastic modulus over F-T cycles.

The test B indicated that the cyclic temperature was repeated for 50 cycles in the range of 5 °C to −20 °C, in accordance with ASTM C 666. After exposed to the freeze-thaw conditions, the compressive strength and elastic modulus tests were carried out with two types of mix designs, C40-2 and C40-4. In Table 14, as the freeze-thaw cycles increased, the compressive strength and elastic modulus decreased because the volume expansion of ice crystal in the pores induced the microcracks under a low temperature [7,20]. Thus, the mechanical properties of the C40-4 mixture were better than those of the C40-2 mixture, in terms of the reduction rate of compressive strength and elastic modulus. The reduction rate of normalized mechanical properties was shown in Figure 12. In detail, the strength reduction was about 10%, and in the case of elastic modulus, the reduction was about 5% less.

### 3.3. Mock-Up Test for Semi-Adiabatic Temperature Monitoring

#### 3.3.1. Preparation of Mock-Up Specimen

**Figure 13:**
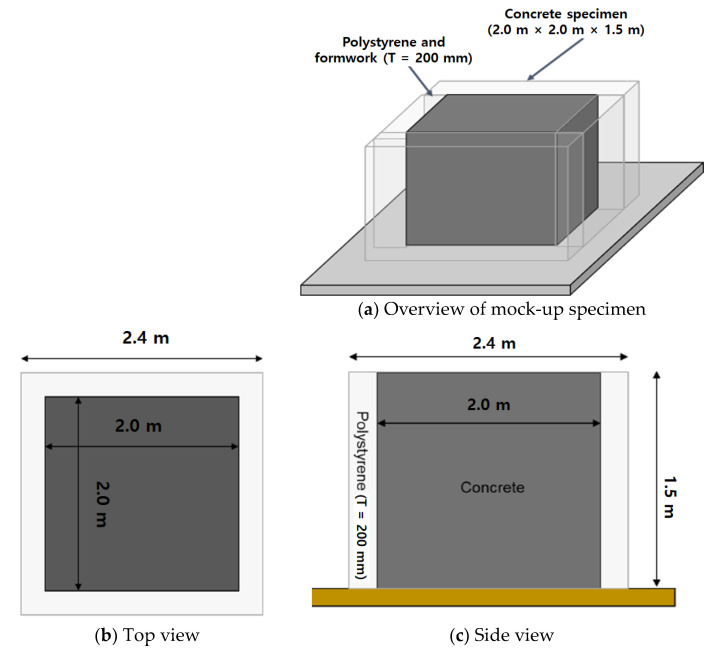
Dimension of Mock-up specimen.

**Figure 14:**
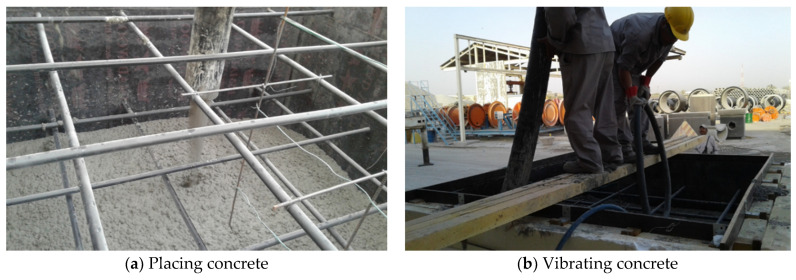
Placing and vibrating of concrete.

A mock-up test was performed to verify the heat of hydration of mass concrete. The two numbers of mock-up specimens were casted with the best optimum mixes for cryogenic concrete such as C40-2 and C40-4. Detailed sizes of Specimens were shown in Figure 13. The size of mock-up specimen was 2.0 m by 2.0 m by 1.5 m and the side surface of it was surrounded with insulation board of 200 mm thick. Concrete placing work and casting specimen had been taken for 30 min after produced. The vibrating works were carefully applied for good consolidation of poured concrete during concrete placing as shown in Figure 14. Temperature of fresh concrete was measured in accordance with AASHTO T 309 [56]. The initial and 30 min temperatures of concrete were controlled less than 32 °C because it was produced in summer season. Table 15 indicated that the slump (flow) and air content of both C40-2 and C40-4 mixtures were satisfied on the target requirement. A wooden form and polystyrene insulation were removed in 21 days after concrete placement. The top surface of concrete had cured with the moisture curing method such as wet blankets and plastic films.

#### 3.3.2. Semi-Adiabatic Temperature Monitoring

**Figure 15:**
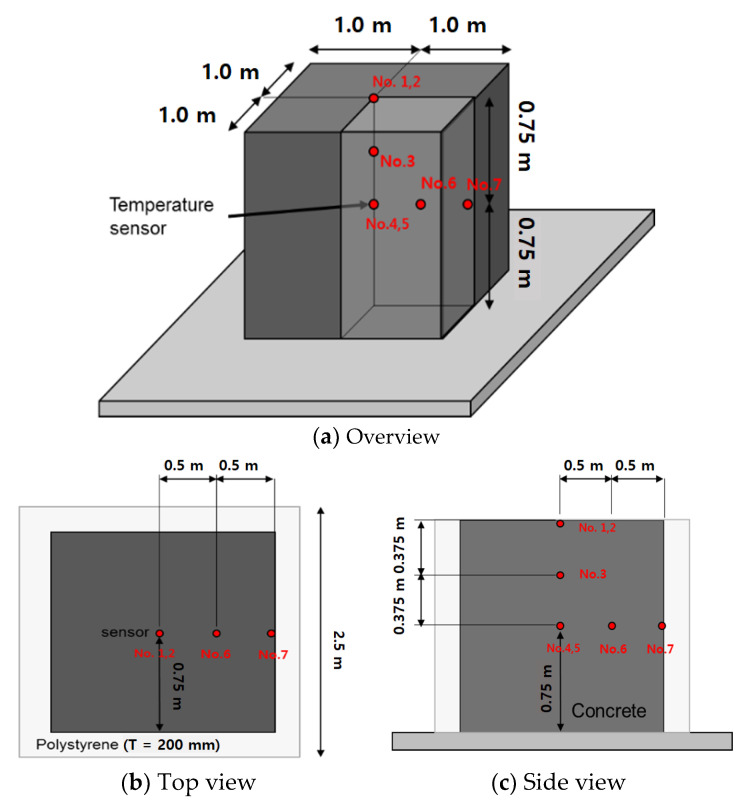
Locations of temperature sensors.

All temperature sensors were installed and mock-up test was pretested before concrete pouring. A total of five temperature sensors (if required, two more spare sensors at the surface and center of specimens) were installed and positioned in concrete specimen at various depths and locations as shown in Figure 15. Temperatures of concrete specimen were measured every 30 min for the first 48 h and then every 1–2 h for 21 days. Ambient temperature was also recorded.

**Figure 16:**
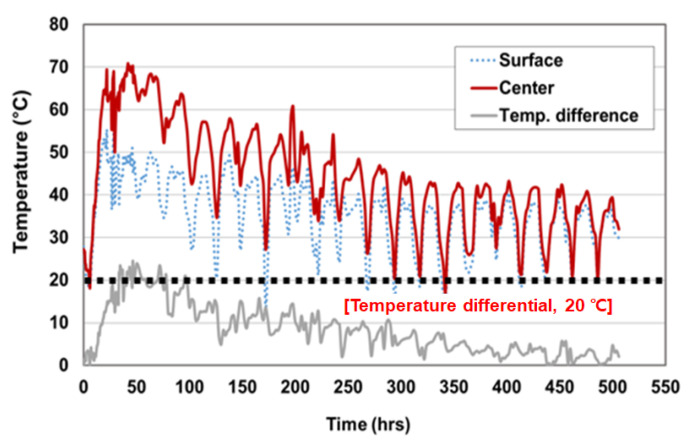
Cryogenic concrete with 65% GGBS and naphthalene type admixture.

**Figure 17:**
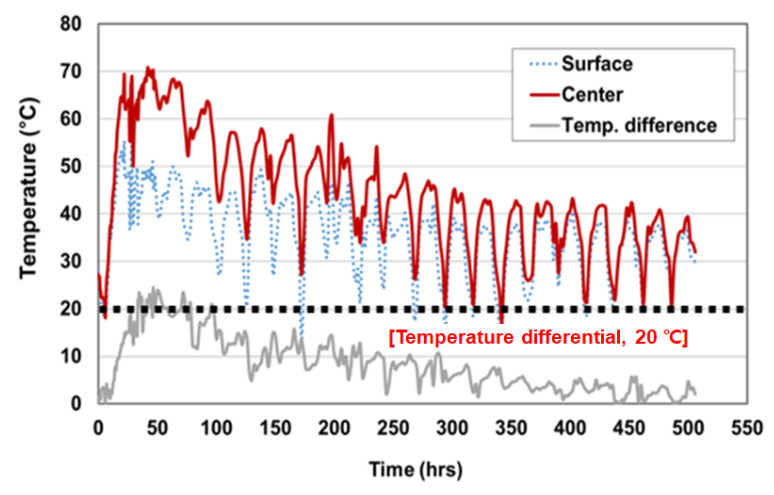
Cryogenic concrete with 65% GGBS and Polycarboxylate type admixture.

The maximum temperature was captured in the center of the mock-up specimen and the temperature difference was measured in the center and surface of mock-up specimen. The maximum temperature of the hardened mass concrete usually occurred between 1 to 3 days after placement and then gradually decreased. According to ACI 308-16, ACI 207.1R and CS 163, in the case of blended cement, the maximum temperature should be controlled in the range of 70 °C and 85 °C, and the temperature difference should not exceed 19 °C [57,58,59]. Table 16 and Figure 16 and Figure 17 show the measured temperature data. For C40-2 with Daracem 208 (naphthalene), the maximum temperature of the center location was 70.85 °C and the temperature difference between center and side surface was 21.85 °C. For C40-4 with Baxel PC 650, the maximum temperature of the center location was 70.8 °C and the temperature difference between center and side surface was 16.95 °C. The maximum temperatures of C40-2 and C40-4 were controlled by less than 75 °C, but the temperature difference of C40-2 did not satisfy the requirement with the exceed of 19 °C. Moreover, the binder amount of the C40-2 and C40-4 mixtures was applied with 495 kg/m^3^ and 475 kg/m^3^, respectively. That meant that the amount of binder for the C40-2 mixture was 20 kg/m^3^ more than that of C40-4. With regard to the heat of hydration, C40-4 mixture was better to control the thermal cracks. Thus, the mix design of C40-4 (GGBS 65% with Baxel PC650) was more suitable for cryogenic concrete, in terms of workability, mechanical and thermal properties under cryogenic conditions and heat of hydration.

## 4. Conclusions

The purpose of this study is to suggest the optimum mix design with a high volume of GGBS replacement and the procedure of the cryogenic test to consider mechanical and thermal properties, and durability performance.

Above all, many research efforts including ACI 376 were reviewed to define the investigation items about mechanical and durability properties under cryogenic environment. Following this, all raw materials were tested to compare the test results with requirements. Particularly in this study, to the control of heat of hydration, the high-volume of GGBS replacement was adopted. For the improvement of freeze-thaw resistance, air entrainer admixture was used. With respect to the emergency condition such as LNG leakage, two types of cryogenic test methods were employed under one-cycle cryogenic condition (Test A) and 50-cycles cryogenic condition (Test B). Next, a mock-up test was conducted to find out the productivity and semi-adiabatic properties. The test results were summarized as below:

(1) With raw materials satisfied the requirements, four mix designs were suggested. To decide the optimum mix design, the slump and retention time of fresh concrete were investigated and the compressive strength of hardened specimens was measured. In this process, with respect to the workability of fresh concrete, C40-2 (GGBS 65% with Daracem 208) and C40-4 (GGBS 65% with Baxel PC650) were better than C40-1 (GGBS 50% with Daracem 208) and C40-3 (GGBS 60% with Baxel PC650). In view of the development of compressive strength, C40-2 and C40-4 were superior to C40-1 and C40-3. In the next step for cryogenic tests, the C40-2 and C40-4 mixture were selected.
(2) After one cycle of cryogenic temperature, the compressive strength and elastic modulus of the C40-2 and C40-4 mixtures tended to be decreased over time, because of the volume expansion of ice crystals contained in the capillary pores. In addition, the degree of water saturation had a significant effect on the frost resistance of the concrete mix.
(3) After exposed to the 50-times freeze-thaw cycles, the compressive strength and elastic modulus tests were carried out the mechanical properties of the C40-4 mixture (GGBS 65% with Baxel PC650) were better than those of the C40-2 mixture (GGBS 65% with Daracem 208), in terms of the reduction rate of compressive strength and elastic modulus. In detail, the strength reduction rate was about 10%, and in the case of elastic modulus, the reduction was about 5% less.
(4) The maximum temperatures of C40-2 and C40-4 were controlled by less than 75 °C, but the temperature difference of C40-2 did not satisfy the requirement with the exceed of 19 °C. Moreover, the binder amount of the C40-2 and C40-4 mixtures was applied with 495 kg/m^3^ and 475 kg/m^3^, respectively. That meant that the amount of binder for the C40-2 mixture was 20 kg/m^3^ more than that of C40-4. With regard to the heat of hydration, the C40-4 mixture was better to control the thermal cracks.

Thus, the mix design of C40-4 (GGBS 65% with Baxel PC650) was more suitable for cryogenic concrete, in terms of workability, mechanical and thermal properties under cryogenic conditions and heat of hydration. This test procedure would be helpful to select the better cryogenic mix design and to define the trend of mechanical, thermal and durability properties and test methods. In the future, the long-term performance of cryogenic concrete needs to be investigated.

## Figures and Tables

**Table 1:** Concrete Materials and Standards [30,31,32,33,34,35].

Materials	Related Standard
Cements	ASTM C 150
Mineral Admixtures (GGBS)	ASTM C 989
Water	ASTM C 94
Fine Aggregate	ASTM C 33
Coarse Aggregate	ASTM C 33
Chemical Admixture (HWRA)	ASTM C 494
Air Entraining Admixture	ASTM C 260

**Table 2:** Properties, requirements and test results for cement according to ASTM C 150 [30].

Cement—Type I Properties	Requirements	Test Result
Magnesium oxide (MgO)	Max. 6.0%	1.22%
Sulfur trioxide (SO_3_)	Max. 3.0%	1.67%
Loss on ignition	Max. 3.0%	1.09%
Insoluble residue	Max. 0.75%	0.19%
Equivalent alkalies (Na_2_O + 0.658K_2_O)	Max. 0.6%	0.52%
Fineness (Air Permeability)	Min. 260 m^2^/kg	315.7 m^2^/kg
3 days Compressive strength	Min. 12 MPa	23.2 MPa
7 days Compressive strength	Min. 19 MPa	36.7 MPa
Time of setting—initial	Not less than 45 min	135 min
Time of setting—final	Not more than 375 min	170 min
Compressive strength—28 days	Min. 28 MPa	46.4 MPa

**Table 3:** Properties, requirements, and test results for GGBS according to ASTM C 989.

Properties	Requirements	Test Results
Amount retained when wet screened on a 45-μm (No. 325) sieve	Max. 20%	8.7%
Fineness by air permeability	No limit	546.7 m^2^/kg
Air content of slag mortar	Max. 12%	6.5%
Activity Index—Grade 100—7 day	Min. 75%	93.2%
Activity Index—Grade 100—28 day	Min. 95%	109.1%
Sulfide sulfur (S)	Max. 2.5%	0.024%
Sulfate (SO_3_)	N/A	0.97
Magnesium Oxide (MgO)	N/A	7.51
Acid soluble chloride ion content	N/A	0.01

**Table 4:** Properties, requirements and test results for fine aggregates [36,37,38,39,40,41,42].

Properties	Requirements	Test Results	Related Standard
Clay lumps and friable particles	Max. 1.0%	0.5%	ASTM C 142
Coal and lignite	Max. 0.25%	0.2%	ASTM C 123
Material finer than 75-um	Max. 3.0%	2.7%	AASHTO T 11
Organic Impurities	Lighter than that of reference standard Color Solution		ASTM C 40
Specific gravity on saturated surface-dry basis	Min. 2.6 g/cm^3^	2.613	ASTM C 128
Water soluble chloride ion content	Max. 0.01%	0.0096%	BS 812:Part 117
Acid soluble sulphate content as SO_3_	Max. 0.3%	0.0553%	BS 812:Part 118

**Table 5:** Properties, requirements and test results for coarse aggregates [36,43,44,45,46,47,48].

Properties	Requirements	Test Results 20 mm	Test Results 10 mm	Related Standard
Clay lumps and friable particles	Max. 1.0%	Nil	Nil	ASTM C 142
Specific gravity on saturated surface-dry basis—calcareous	Min 2.65 g/cm^3^	2.688	2.680	ASTM C 127
Water absorption	Max. 1.0%	0.5%	0.6%	AASHTO T 85
Los Angeles loss	Max. 30%	21.2%	21.8%	AASHTO T 96
Acid soluble chloride ion content	Max. 0.01%	0.0085%	0.0081%	AASHTO T 260
Acid soluble sulphate content as SO_3_	Max. 0.4%	0.0115%	0.0168%	BS 812:Part 118
Soundness using Sodium sulphate	Max. 12%	2.4%	2.5%	ASTM C 88
Alkali reactivity	Max. 0.04%	Innocuous	Innocuous	ASTM C 1293

**Table 6:** Properties, requirements and test results for mixing water.

Properties	Requirements	Test Results
PH Value	6.0–8.0	7.0
Residue Content	6.0–7.6%	6.8%

**Table 7:** Trial mix proportions of cryogenic concrete.

No.	W/B (%)	Unit Weight (kg/m^3^)							Admixture (Liter)		
		Water	Binder		Coarse Agg.			Fine Agg.	Type I	Type II	AE
			Total	OPC	GGBS	20 mm	10 mm	Sand			
C40-1	28	131	490	245	245	690	480	580	9.0–11.0	-	0.25–1.5
				(50%)	(50%)						
C40-2	28	133	495	175	320	680	470	580	9.0–11.0	-	0.25–1.5
				(35%)	(65%)						
C40-3	28	128	475	190	285	690	480	610	-	6.0–7.0	0.25–1.5
				(40%)	(60%)						
C40-4	28	128	475	166	309	690	480	610	-	6.0–7.0	0.25–1.5
				(35%)	(65%)						

Type I: Naphthalene type (Daracem 208), Type II: Polycarboxylate type (Baxel PC 650).

**Table 8:** Test method and concrete specimens.

No.	Fresh Concrete		Hardened Concrete				Reserved Samples	Total No. Samples
	Air Content (%)	Slump (mm)	Compressive Strength (MPa)					
			1 d	3 d	7 d	28 d		
C40-1	4 ± 1.5	Initial 30 min. 60 min. 90 min. 120 min	3	3	3	3	3	15
C40-2			3	3	3	3	3	15
C40-3			3	3	3	3	3	15
C40-5			3	3	3	3	3	15

**Table 9:** Test method and concrete specimens under cryogenic condition.

Test A Method—One-Time Cryogenic Cycle	Curing (Day)	Temperature Conditions			
		Ambient (0 min)	−50 °C (15 min)	−120 °C (30 min)	−196 °C (60 min)
Compressive strength	28	3	3	3	3
Tensile strength	28	3	-	-	3
Elastic modulus	28	3	3	3	3
Thermal expansion coefficient	28	2	-	-	2
Thermal conductivity	28	2	-	-	2
Total	-	13	6	6	13

**Table 10:** Test method and concrete specimens after 50 freeze-thaw cycles.

Test B Method—50-Times Freeze/Thaw Cycles	Temperature Conditions		
	Curing (Day)	Ambient	5–−20 °C
Compressive strength after cycling	28	3	3
Elastic modulus after cycling	28	3	3
Total		6	6

**Table 11:** Test results of compressive strength, elastic modulus, poisson ratio and absorption.

Test Item	Immersed Time (min.)	Compressive Strength (MPa)	Elastic Modulus (GPa)	Poisson Ratio	Absorption (%)
C40-2	0 min. (ambient)	61.8	36.1	0.1483	3.57
	15 min.	52.8	35.1	0.1440	3.50
	30 min.	53.9	34.2	0.1437	2.28
	60 min.	53.9	33.9	0.1440	2.40
C40-4	0 min. (ambient)	62.17	39.8	0.1517	2.93
	15 min.	60.0	35.8	0.1437	2.17
	30 min.	57.9	35.7	0.1430	2.23
	60 min.	57.5	35.4	0.1437	2.21

**Table 12:** Test results of length change and splitting tensile strength.

Test Item	Immersed Time (min.)	Length Change (mm)	Splitting Tensile Strength (MPa)
C40-2	0 min. (Ambient)	0.0015	4.30
	60 min.	−0.063	3.44
C40-4	0 min. (Ambient)	0.0002	4.43
	60 min.	−0.0690	3.21

**Table 13:** Test results of thermal conductivity.

Test Item	Temperature (°C)	Thermal Conductivity (W/m∙K)
		300 mm × 300 mm × 90 mm
C40-2	20 °C	1.512
	−160 °C	0.643
C40-4	20 °C	1.485
	−160 °C	0.723

**Table 14:** Test results of compressive strength, elastic modulus, poisson ratio and absorption.

Test Item	Freezing Thawing (Cycle)	Compressive Strength (MPa)	Elastic Modulus (GPa)	Poisson Ratio	Absorption (%)
C40-2	0	59.2	38.1	0.147	2.94
	50	53.3	36.5	0.142	3.42
C40-4	0	66.0	42.2	0.156	2.69
	50	60.7	41.5	0.150	3.01

**Table 15:** Test result of fresh concrete produced from batch plant.

Test Item	Temperature (°C)		Slump (Flow) (mm)		Air Content (%)		Density (kg/m^3^)	
	Initial	30 Min.	Initial	30 Min.	Initial	30 Min.	Initial	30 Min.
C40-2	26.0	27.1	240	220	5.5	5.2	2303	2369
C40-4	28.9	29.2	650	620	4.5	4.0	2386	2393

**Table 16:** Results of measured temperature data (replacement of GGBS 65%).

No.	Admixture	Temp. Max. at Center (°C)		Temp. Difference (°C)
		Surface	Center	
C40-2	Daracem 208	49.00	70.85	21.85
C40-4	Baxel PC 650	53.85	70.80	16.95

## Data Availability

Data sharing is not applicable to this article.
